# Curved puncture technique using a flexible stainless-steel needle in endoscopic ultrasound-guided hepaticogastrostomy

**DOI:** 10.1055/a-2480-3803

**Published:** 2024-12-04

**Authors:** Haruo Miwa, Yuichi Suzuki, Shotaro Tsunoda, Kazuki Endo, Ritsuko Oishi, Hiromi Tsuchiya, Shin Maeda

**Affiliations:** 1 26437 Gastroenterological Center, Yokohama City University Medical Center, Yokohama, Japan; 2 26438 Gastroenterology, Yokohama City University School of Medicine Graduate School of Medicine, Yokohama, Japan

Puncturing the intrahepatic bile duct of segment 2 (B2) in an endoscopic ultrasound-guided hepaticogastrostomy (EUS-HGS) can be challenging when the bile duct is only accessible through the transesophageal route 1 2 . In such cases, a flexible stainless-steel needle (SonoTip Pro Control 22G; Medi-Globe, Rosenheim, Germany) may provide a wider range of puncture angles 3 . We report a case of EUS-HGS performed using a 22-gauge stainless-steel needle in a patient in whom B2 puncture was challenging ( Video 1 ).

Endoscopic ultrasound-guided hepaticogastrostomy is performed using a 22-gauge stainless-steel needle with a curved puncture technique.Video 1

A 73-year-old man with a benign hilar biliary stricture and refractory cholangitis due to hepatic stones was referred to our institution ( Fig. 1 ). Transpapillary biliary stenting was attempted for both B2 and B3; however, the stent could not be placed over a hepatic stone in B2 ( Fig. 2 ). Because the patient was readmitted with segmental cholangitis, EUS-HGS for B2 was performed. On ultrasound images, the dilated B2 duct was only detected over the landmark clip at the esophagogastric junction. An initial attempt to puncture the nondilated bile duct showed that the needle pathway was positioned above the landmark. EUS images from the stomach revealed that B2 was located in a deeper area. It was considered that the cobalt–chromium needle, because of its straight trajectory, would not be able to puncture the B2 branch. Therefore, the 22-gauge stainless-steel needle was chosen. Using the full angulation of the up-angle and elevator system, the needle tip was strongly curved, facilitating the successful puncture of B2 ( Fig. 3 ). After contrast injection had been performed, a 0.018-inch guidewire was advanced, followed by insertion of an ultra-tapered catheter. The guidewire was replaced with a 0.035-inch wire and a plastic stent was successfully placed ( Fig. 4 ). The patient was discharged 3 days after the procedure, without experiencing any complications.

**Fig. 1:**
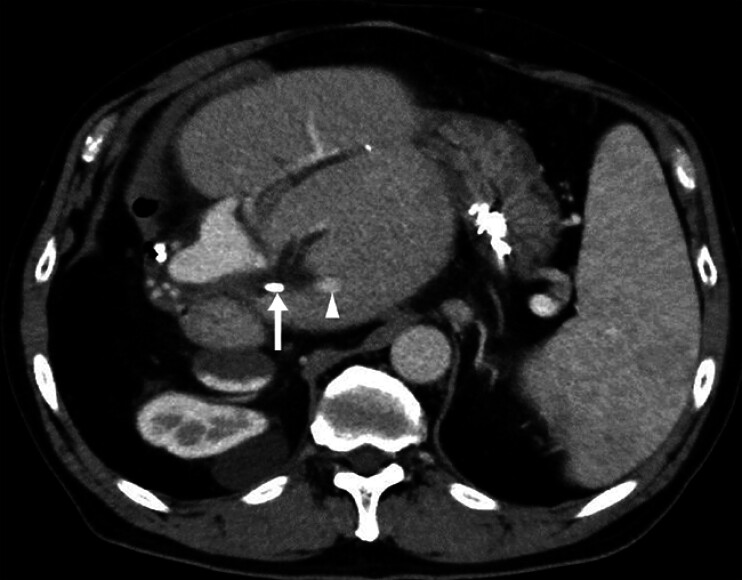
Computed tomography image showing a hepatic stone in segment 2 (arrowhead). The proximal tip of the biliary stent is located downstream of the stone (arrow).

**Fig. 2:**
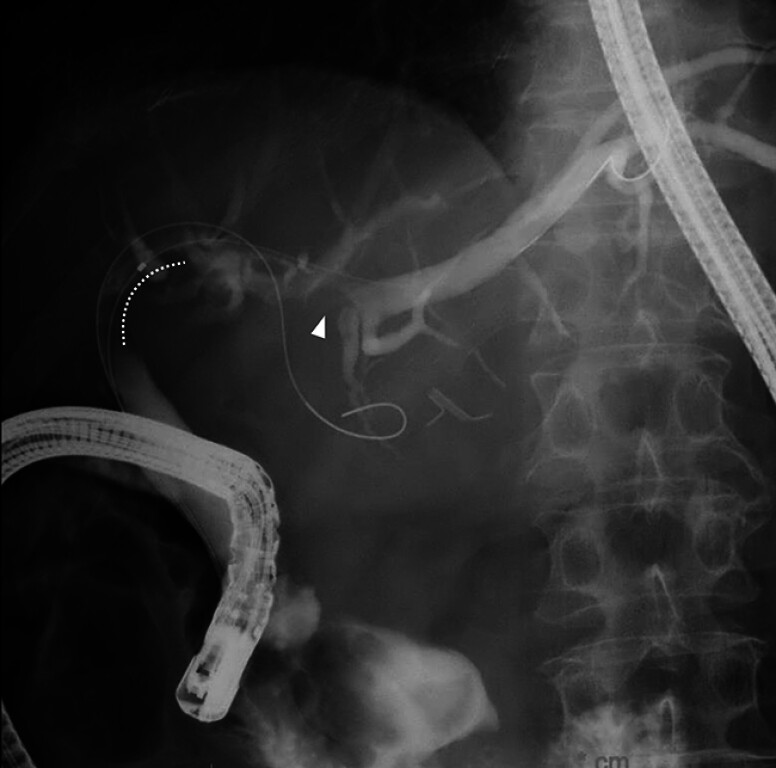
Fluoroscopic image during endoscopic retrograde cholangiopancreatography showing a biliary stricture at the perihilar lesion (dotted line) and a contrast defect due to the hepatic stone in the periphery of B2 (arrowhead).

**Fig. 3:**
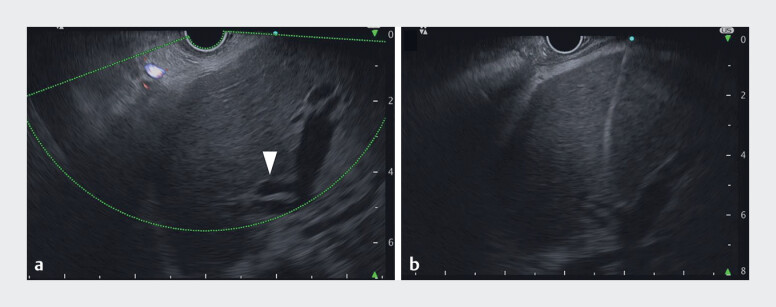
Endoscopic ultrasound (EUS) images during EUS-guided hepaticogastrostomy showing: **a** a branch of B2 in an area deeper than 4 cm (arrowhead); **b** the B2 branch being punctured vertically using the curved puncture technique.

**Fig. 4:**
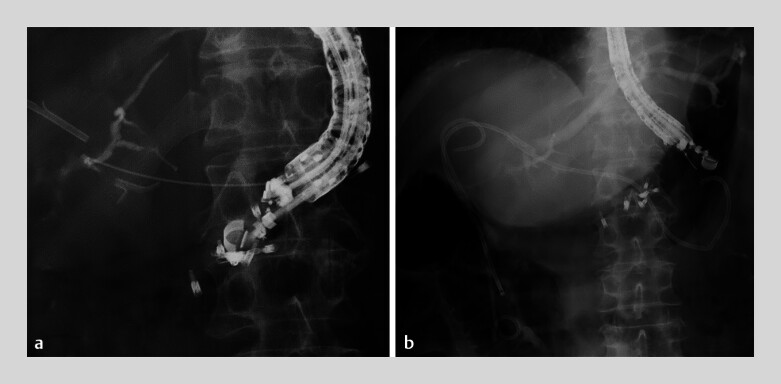
Fluoroscopic images during endoscopic ultrasound-guided hepaticogastrostomy showing: **a** a branch of B2 being punctured using a 22-gauge stainless-steel needle; **b** successful placement of a plastic stent.

To the best of our knowledge, this is the first report of a curved puncture technique using a 22-gauge stainless-steel needle, which potentially expands the indications for EUS-HGS in challenging cases.

Endoscopy_UCTN_Code_TTT_1AS_2AH

## Notes

Endoscopy E-Videos https://eref.thieme.de/e-videos
*E-Videos* is an open access online section of the journal *Endoscopy* , reporting on interesting cases and new techniques in gastroenterological endoscopy. All papers include a high-quality video and are published with a Creative Commons CC-BY license. Endoscopy E-Videos qualify for HINARI discounts and waivers and eligibility is automatically checked during the submission process. We grant 100% waivers to articles whose corresponding authors are based in Group A countries and 50% waivers to those who are based in Group B countries as classified by Research4Life (see: https://www.research4life.org/access/eligibility/ ). This section has its own submission website at https://mc.manuscriptcentral.com/e-videos .
